# In pursuit of objective dry eye screening clinical techniques

**DOI:** 10.1186/s40662-015-0032-4

**Published:** 2016-01-18

**Authors:** Anastasios John Kanellopoulos, George Asimellis

**Affiliations:** Laservision.gr Clinical and Research Eye Institute, 17 Tsocha Street, Athens, 11521 Greece; Department of Ophthalmology, New York University Medical School, New York, NY USA

**Keywords:** Dry eye, Shrimer’s test, TBUT test, Inflammation, OCT, Epithelial thickness, Anterior-segment

## Abstract

Dry eye is a multifactorial, progressive, and chronic disease of the tears and ocular surface. The disease is multi-factorial and has intermittent symptoms. Discomfort, visual disturbance, tear film instability with potential damage to the ocular surface, and increased tear film osmolarity are known associates.

Dry eye is a common clinical problem for eye-care providers worldwide and there is a large number of clinical investigative techniques for the evaluation of dry eye. Despite this, however, there is no globally accepted guideline for dry eye diagnosis and none of the available tests may hold the title of the ‘gold standard’. The majority of the techniques involved in the diagnosis of the disease, particularly for its early stages, has a large degree of subjectivity.

The purpose of this article is to review existing dry eye investigative techniques and to present a new objective dry eye screening technique based on optical coherence tomography.

## Background

Dry eye disease [1] is responsible for major population morbidity and considerable economic impact in terms of both direct and indirect costs [2] because of the disease’s progressive nature and the significant toll on quality of life [3]. In addition, it may present major challenges in a refractive surgery candidate assessment [4]. Its condition may range from mild/episodic to severe/chronic: the disorder can be manifested with many symptoms including visual disturbance (blurred and fluctuating vision), foreign-body sensation and eye discomfort (patient-reported), irritation, ocular surface inflammation, redness, excess tearing, and photosensitivity [5–9].

Contributing factors to dry eye may be classified as ocular, medical, pharmaceutical, iatrogenic, environmental, and contact lens wear [10].

Ocular conditions include eyelid (blepharitis) and ocular surface inflammation, and chemical burns. Medical conditions include Sjögren’s syndrome, [11] vitamin-A and omega-3 fatty acid deficiency, rheumatoid arthritis and other rheumatologic diseases, as well as diabetes and thyroid problems. Reactions to certain medications such as antihistamines, diuretics, sleeping pills, decongestants, blood-pressure medications and antidepressants, postmenopausal estrogen therapy medications, and isotretinoin-type drugs for acne treatment, may contribute in their capacity to impact tear production [12]. Iatrogenic conditions include eyelid/facial surgery and corneal refractive surgery. Specific to laser in situ keratomileusis (LASIK), although pre-existing dry eye may be subclinical, a sizable portion of LASIK patients may develop reduced basal tear flow, [13] attributed to surgical severing of the nerves by the creation of the LASIK flap, [14, 15] and/or by the excimer laser ablation [16]. Environmental conditions include aridity, cold/windy air, and repetitive occupational tasks that require increased concentration that affect blinking [17].

## Review

### Clinical investigative techniques for the evaluation of dry eye

The importance of proper and timely distinction between healthy and affected eyes is unquestionable [18–20].

There is a large number of clinical investigative techniques for the evaluation of dry eye. The current options include slit-lamp observations, tear film stability assessment (invasive/non-invasive tear-film breakup time (TBUT) measurement, tear film interferometry), [21] tear secretion assessment tests (Schirmer lacrimation with or without anesthesia, thread methods), tear clearance assessment (fluorescein clearance test, tear function index, fluorophotometry), [22] ocular surface damage assessment (corneal and conjunctival, rose Bengal, lissamine green staining, cytology), [23] lipid layer assessment (precorneal/meibomian grading), [24, 25] tear osmolarity tests, [26–29] and patient subjective symptom questionnaires [30].

However, the problem of definite dry eye assessment is bedeviled by many parameters and several aspects may make a safe diagnosis challenging particularly in the early or mild stages. Poor diagnostic test repeatability [31] that is manifested as significant false-positive/negative rates, [32] broad range of variability, wide range of sensitivity and specificity, and dependence on clinical conditions [33] are some of the reasons cited [34].

We also have to consider the multi-factorial nature of the disease and the intermittency of the symptoms: there is a continuum of susceptibility and possible overlay/interference of the presented symptoms with other ocular irritations and environmental influences [35]. Seasonal and diurnal variations are also factors that may affect symptoms [36].

Another aspect is that among the prevailing investigative techniques such as Schrimer’s lacrimation and TBUT tests, there are examiner subjectivity, [37] external stimuli influence, [38] and accurate documentation difficulty [39]. The same is true for investigative techniques based on patient-reported symptom questionnaires [40–42]. Published evidence suggest that clinical dry eye symptoms alone may be insufficient for proper diagnosis of the disease [43, 44].

Adding to the challenge is the fact that despite the existence of several dry eye scoring systems, [45] there is no globally-accepted guideline for dry eye diagnosis and none of the available tests may hold the title of the ‘gold standard’ [46]. With no widely accepted gold standard against which to measure the tests, manipulation of the diagnostic criteria used for the standard can affect the reported sensitivity of new tests.

The other end of the problem is that there is no established clear-cut threshold for early-stage dry eye definite assessment. This aspect hinders the adoption of cut-off values for any traditional metric. The problem of establishing precise cut offs between normal and dry eye patients lies less with the test and more with the understanding that like almost all diagnostic tests, not every individual has the same threshold for revealing disease but rather there is a range in the population; one that the clinician should have a clear understanding of in regards to disease development. Notwithstanding that, cut offs with highly useful clinical utility are available and supplementary values e.g., inter-eye differences in tear osmolarity, add to the specificity of the tests.

There is a critical need therefore, for a consensus of newer/updated investigative techniques and metrics that will better reflect the differential discrimination of the disease [33].

### In search for an objective dry eye assessment technique

A novel objective investigative technique for dry eye screening that has been recently proposed by our team is the objective evaluation of epithelial thickness by anterior-segment optical coherence tomography (AS-OCT) [47]. Specifically, overall epithelial thickness may reflect conditions such as moderate or even subclinical dry eye and may aid in the timely diagnosis.

### Why epithelium?

It has been established that the epithelial layer thickness and morphology may be influenced by hypoxia, [48] contact lens wear, [49, 50] corneal ectasia, [51] corneal cross-linking, [52] and ocular surgery such as clear-cornea incision cataract removal, [53] corneal lamellar surgery, [54] and corneal refractive surgery [55, 56].

Epithelial thickening may be an alarming indication for corneal abnormality. In a previous investigation of the three-dimensional epithelial thickness in keratoconic eyes, [51] we identified an overall thicker epithelium that might be a result of a reactive process; the epithelium appears to thicken in less ‘rigid’ corneas due to being more susceptible to mechanical variations produced by one or a combination of factors including eye rubbing and increased blinking mechanism [57].

Regarding dry eye, advanced stages may be reflected as morphological epithelial damage [58]. Increased epithelial thickness has been associated with dry eye in a rat model, and has been associated with the inflammatory process [59]. Atopic keratoconjunctivitis has been associated with significant alterations of the basal epithelium, and subbasal and stromal corneal nerves, related to the changes in tear functions and corneal sensitivity [60]. Studies with scanning microscopy have identified altered central epithelial thickness in dry eyes [61] or epithelial thickness irregularities in cases with Sjögren’s syndrome [62, 63]. In a confocal laser scanning microscopy study, [64] the mean superficial and intermediate epithelial cell densities in the central cornea in the dry eye groups were significantly lower than in normal participants. Dry eye corneas showed significant corneal epithelial alterations, presumably due to increased desquamation of the superficial cell layer.

### The case for OCT

In the past, the available investigating and clinical evaluation modalities for the purpose of epithelial thickness imaging have been high-frequency ultrasound (HF-UBM), time-domain OCT, and confocal microscopy through focusing (CMTF) [65]. None of these were fully clinically applicable and/or had a commercially available model for this specific use. For example, HF-UBM employs fluid coupling, which explains why we have not identified any reports on dry eye and HF-UBM measurements. CMTF requires instrument interface contact with the cornea, and had been restricted in this application due to the degraded precision by eye movement during the long acquisition time. Other available techniques are either invasive or require contact between the probe and the ocular surface, and thus cannot provide precise *in vivo* measurement of the entire epithelial thickness.

OCT has the advantage of the ease of *in vivo* non-contact application and speed of optical imaging [66]. Until recently, however, its application in epithelial thickness imaging involved either investigator-modified software/hardware [67–69] or caliper software measurement techniques [58, 70] (for example, by manually placing cursors to measure epithelial thickness in each location).

**Fig. 1:**
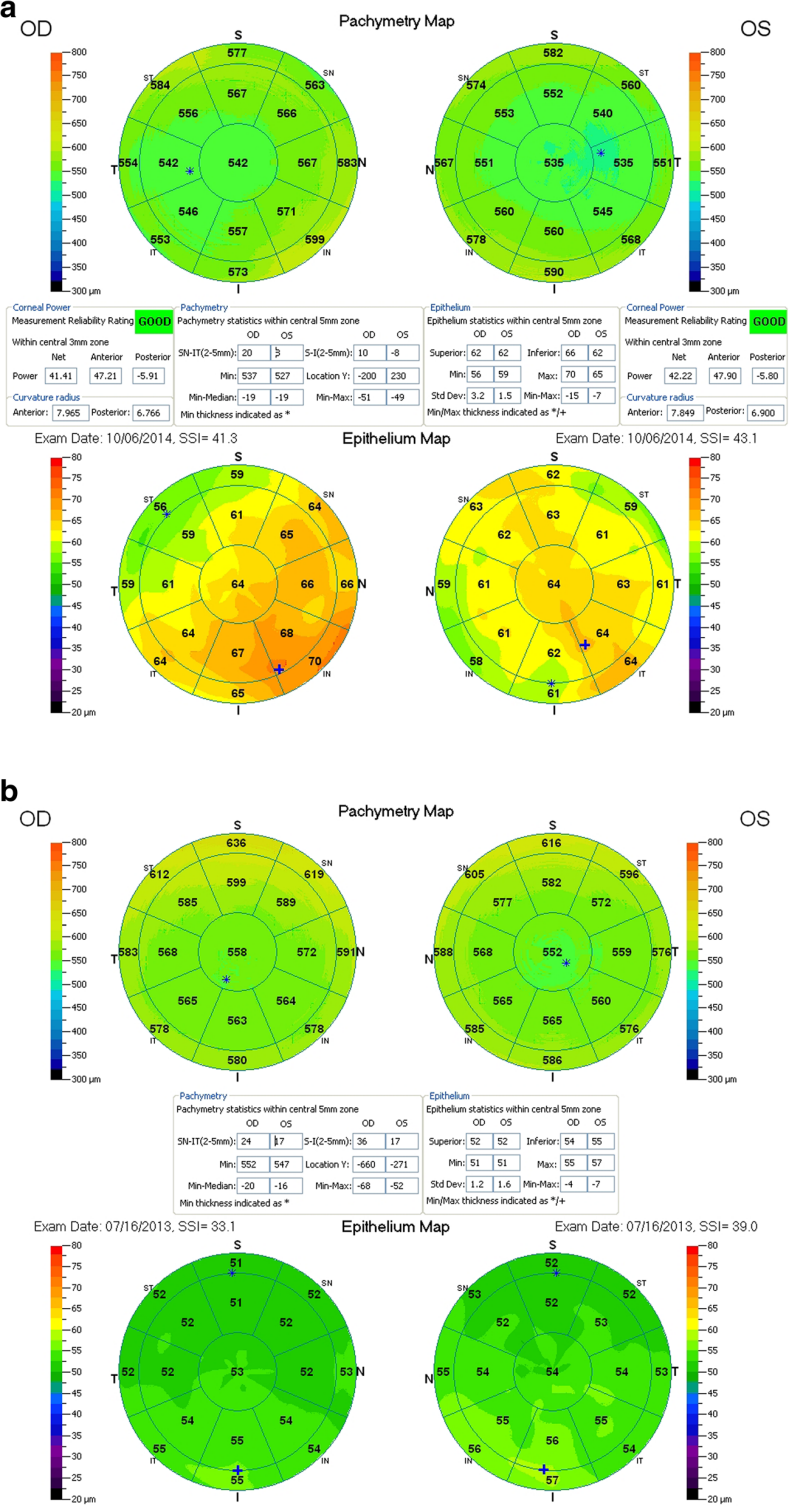
Representative thickness maps provided by the OCT system report, including total corneal, and corneal epithelium thickness maps. *Top*-**a** is a typical example from a ‘normal-group’ patient, while *bottom*-**b** from a dry-eye patient

The novelty offered by the Fourier-domain anterior-segment OCT system RtVue-100 (Optovue Inc., Fremont, CA) is that it is the first OCT system that incorporates epithelial thickness maps (extending, currently up to 6-mm diameter) analysis. In each meridional scan, the system software automatically identifies the air-tear film interface and the epithelium-Bowman layer interface. The report then provides the pachymetry maps of both total corneal and corneal epithelial thickness shown in Fig. 1. This screening examination may be included in the routine screening protocol of all patients [71]. Thus, this examination potentially presents a practical clinical tool for qualitative (by evaluation of the three-dimensional epithelial thickness mapping) and quantitative epithelium evaluation (data for absolute average, central, and peripheral epithelial thickness measurements). This investigation revealed that the measurement repeatability was of the order of 1 μm, and the topographic thickness variability was found to be of the order of 0.25 μm [71]. There were some epithelial thickness differences between male and female groups (Female group average 52.58 ± 3.19 μm, Male group average 54.10 ± 3.34 μm). Topographic thickness variability between the two groups did not differ at the 0.05 level of significance (p = 0.173). Age also appeared to be an influencing factor: epithelial thickness for the younger group was 52.95 ± 3.44 μm, while for the older group was 53.64 ± 3.21 μm (not statistically significant, however).

OCT epithelial thickness mapping with this system has recently gained clinical impetus and research interest by other teams as well [72, 73].

### A new proposition

In pursuit of an objective, repeatable, and quantitative clinical test that may aid in the differential diagnosis of dry eye, we introduced the concept of corneal epithelial thickness as a possible tool in dry eye assessment, and reported initial clinical results regarding three-dimensional corneal epithelial thickness mapping in dry eye corneas employing the RtVue-100 OCT system [47].

Our study suggested that there is a statistically significant thicker corneal epithelium in mid-aged female population diagnosed with moderate dry eye in comparison to an age-matched control population.

We believe that the clinical difference observed might play a role in routine screening and treatment assessment, which may precede the specific dry eye measurements that may or may not be part of a standard screening protocol. The findings reported herein may also be very useful in the screening of refractive surgery candidates, and even in the assessment of post-operative iatrogenically induced dry eye [13].

In a recent study by Cui et al. [74] central epithelium thickness in dry eyes, measured by the same Fourier-domain OCT, was found to be significantly thinner than that in normal eyes. Notably, the thinner tendency was larger in the more severe stages. Our team has also identified thinning and increased topographic variability of the epithelium in older age-patients [71]. It is possible that in advanced stages in older-age patients, the chronic insidious injury by a deficient tear film or the destruction of stem cells at the limbus may be likely causes for epithelial thinning. In addition, we emphasize that one should not only take into account the central epithelial thickness, but also information deriving from the overall epithelial thickness, as due to immune and angiogenic privilege, central cornea may be less sensitive to inflammation than the limbus and conjunctiva [75].

Further cell morphology studies in epithelial thickness associated with dry eye i.e., with confocal microscopy, may be warranted to differentiate these noted differences, which may include epithelial hypertrophy/ hyperplasia, swollen cells, and/or increase in the number of cellular layers.

The anticipated clinical ramifications of the application are prospectively very positive since this screening indicator is based on a commercially available instrument that can easily be integrated into daily clinical practice and with increasing clinical screening potential.

### Our investigation

We conducted a comparative retrospective investigation forming two groups. The ‘control’, group A (n = 70 eyes, 35 patients), consisted of ambulatory female patients with unoperated, normal eyes with no ocular pathology other than refractive error, and no dry eye condition, confirmed by a complete ocular clinical evaluation. The ‘dry eye‘, group B (n = 70 eyes, 35 patients), consisted of female patients with clinically confirmed dry eye, otherwise unoperated and with no other ocular pathology save for possible refractive error. All patients signed an informed consent form, releasing anonymous data evaluation for scientific purposes.

Both groups consisted of female patients with dry eye because they compose 10:1 compared to males as observed in our clinical practice (unpublished data). Dry eye was diagnosed via TBUT measurement (dry eye considered if under 5 s) and Schirmer basic lacrimation test (dry eye considered if under 5 mm). Exclusion criteria were anterior basement membrane and other corneal dystrophies, and/or rheumatic diseases. No patient with reported previous use of contact lens nor with recent dispensing of artificial tear drops was enrolled in this study in either group.

For each eye we measured and analyzed statistically within the central 5 mm zone the average, superior, and inferior epithelial thickness, as well as topographic thickness variability, as reported by the standard deviation of the seventeen (17) segments (shown in Fig. 1) local thickness measurements. Average epithelium thickness was computed for each case within the 5 mm zone as the average of the seventeen segments local thickness measurements. Examples of such maps from each group are shown in Fig. 1.

The study suggested an overall thicker epithelium in the group of dry eye female patients, and specifically, a statistically different epithelial thickness between the dry eye and control groups. The differences (average in dry to normal eyes) ranged, for the central thickness by +6.5 μm and for the average thickness by +6.2 μm. Details are reported in [47] and [76].

Despite the overlap in the thickness between control and dry eye epithelial layer thickness, these differences were statistically significant. Moreover, these differences were larger than the repeatability measurement fluctuations. In a recent evaluation [71] of a large population of healthy eyes (373 cases), average epithelial thickness repeatability was at 0.8 ± 0.7 μm.

Increased epithelial thickness may also be encountered in ectatic corneas. The differentiating factor between the thicker ‘dry eye’ and the thicker keratoconic epithelium lies in the topographic thickness variability. In normal eyes, we measured an average of 1.8 ± 1.1 μm [71]. In the dry eye study, the topographic thickness variability was 2.5 ± 1.5 μm, slightly larger than in the ‘healthy eye’ population, while in the keratoconic study thickness variability was found to be significantly larger (up to 10.3 μm), thus enabling differentiation.

In this study, the specific imaging with the RtVue system might also influence dry eye epithelial measurements by the AS-OCT device. In a previous OCT study of epithelial thickness by Francoz et al. [58] with a different instrumentation, difference between central epithelial thickness between middle-aged normal (48.8 ± 3.0 μm) and dry eye population (49.0 ± 4.1 μm) was much smaller. This can be attributed to investigative differences: in the current study, average epithelial thickness was accurately reported on the select meridian scans and interpolated on the space between while the study by Francoz et al. implemented manual position on select scanned meridians to measure epithelial thickness. The different geographical locale might also be a factor.

## Conclusions

Among the newly emerging dry eye testing options, tear film osmolarity may be considered an objective technique. The test employs a disposable test chip (TearLab Corp., San Diego, CA) that collects a small (50 nL) tear sample from the lower meniscus [77]. Analysis is based on electrical impedance (milliosmoles per liter) of the tear sample [28].

Newly emerging techniques that may be considered are thermography, [78] a technique incorporated in a clinical autorefractor/keratometer (RC 5000; Tomey Corporation, Nagoya, Japan), as well as the noninvasive tear breakup time recorded and digital measurement with an optional feature of a corneal Placido-ring topographer (Keratograph 5M; Oculus Optikgeräte GmbH, Wetzlar, Germany) [79].

Sensory testing could also be useful diagnostically, notwithstanding issues relating to altered corneal epithelial barrier function [80, 81].

We introduced and presented a novel dry eye screening technique based on a clinical OCT device. The screening is part of our established protocol not only for dry eye, but also for general evaluation of the cornea; thus the potential for dry eye alert comes as a collateral benefit. We emphasize that the clinical diagnostic capacity of the increased overall epithelial thickness that we introduced has importance for early-stage, subclinical dry eye, and not severe dry eye, for which many other techniques may offer a more concrete diagnosis.
